# Study on the Structure and Properties of Biofunctional Keratin from Rabbit Hair

**DOI:** 10.3390/ma14020379

**Published:** 2021-01-14

**Authors:** Xiaoqing Wang, Zhiming Shi, Qinglong Zhao, Yu Yun

**Affiliations:** 1 School of Materials Science and Engineering, Inner Mongolia University of Technology, Hohhot 010051, China; wxqing.999@163.com; 2 College of Textile and Light Industry, Inner Mongolia University of Technology, Hohhot 010080, China; z15124778361@163.com (Q.Z.); yunyuxz@163.com (Y.Y.)

**Keywords:** keratin, protein structure, biocompatibility

## Abstract

Keratin is widely recognized as a high-quality renewable protein resource for biomedical applications. A large amount of rabbit hair waste is produced in textile industries, because it has high medullary layer content, but poor spinnability. Therefore, it is of great significance to extract keratin from waste rabbit hair for recycling. In this research, an ultrasonic-assisted reducing agent-based extraction method was developed and applied to extract keratin from rabbit hair. The results showed that the ultrasonic treatment had a certain destructive effect on the structure of the fiber, and when combined with reducing agent, it could effectively promote the dissolution of rabbit hair, and extract keratin with high molecular weight between 31 and 94 kDa. The structure and properties of keratin were studied. Compared to the rabbit hair, the cystine content of keratin was significantly reduced, and the secondary structure changed from α-helix to β-sheet. The keratin products show excellent biocompatibility and antioxidant capacity. In addition, large keratin particles can be formed by assembly with a balance between intermolecular hydrophobic attraction as the concentration of urea in keratin solution decreased during dialysis.

## 1. Introduction

Keratin is a type of scleroprotein made up of amino acids that are linked by both intramolecular and intermolecular interactions, including disulfide bonds, ionic bonds, hydrogen bonds, and hydrophobic interaction. Keratin is found widely in human and animal organs, including epidermis, hoof, horn, hairs, feather and protein fiber. However, there are a lot of disulfide bonds in aforementioned materials which are responsible for its chemical stability and high mechanical strength [1], therefore its application has been limited, resulting in a severe waste of resources and major accumulation of environmental pollution. Therefore, the conversion of keratin wastes into useful materials has attracted increasing research attention. Recently, with the investigation of keratin, scholars have found that keratin is a natural biomaterial with excellent biocompatibility and controlled biodegradability for tissue engineering and medical applications [2,3], such as wound healing [4], bone regeneration [5], hemostatic dressings [6], and controlled drug delivery [7].

Keratin can be extracted from the waste fiber by several methods, such as crushing (to produce feed additives), burning, and burying (to produce fertilizers) [8,9,10]. These methods, however, are environmentally unfriendly. To study the recovery and utilization of the fiber waste, to prevent the environmental pollution, and to appropriately utilize the resources, most research mainly concentrate on the extraction and purification of keratin from low quality animal fibers by disrupting disulfide bonds and peptide bonds using chemical substances [11,12]. Main keratin extraction methods (from protein fibers) include physical method (e.g., microwave irradiation) [13], steam explosion method [14], and chemical method (e.g., hydrolysis, oxidation, reduction, and ionic liquids). In the treatment of wool with an alkali solution, the high-concentration alkali solution dissociates hydrogen atoms from sulfate and carboxylic groups, causing damage to the main protein backbone. In the alkali extraction method, high amounts of acid are required to neutralize and precipitate the protein, which is one of the main factors hindering the scale-up of this method [15,16]. The acid leaching method has been introduced to recycle keratin [17,18,19]; however, the efficiency of this method is low, and it also requires a long period of time to hydrolyze fiber. The main reaction in the oxidation-extraction is the transformation of disulfiram to sulfonate [20]. Thiol reagent is first used to extract keratin through reduction method [21]; this is based on the fact that cysteine disulfide bonds (R–S–S–R) is broken, and cysteine (R–SH) is then engaged. In the follow-up studies, sodium sulfide was utilized as a substitute for mercaptoethanol in the extraction of keratin through the streptolysin step, in which cysteine (R–SH) and cysteine-sulfonate ($R-S-SO_{3}^{-}$) were formed [22,23]. The step was conducted along with urea and surfactants; urea is a protein denaturant that can cause the swelling of keratin structure by weakening the hydrophobic interactions within the polypeptide chain and facilitating the effect of the reducing agent on the polypeptide chain [24]. The generated cysteine is unstable and can be easily oxidized by air. The micellar conformation between surfactants and proteins has been reported to be able to prevent oxidization. Recently, L-cysteine has been used as a reducing agent to extract keratin from rabbit hair [25]. Ionic liquids, which have high solvation for specific solutes, have been widely used for the extraction of keratin, and the solubility of wool in different ionic liquids has been evaluated [26,27]. A novel, eco-friendly and benign choline chloride/oxalic acid, which is a deep eutectic solvent, has been applied to dissolve rabbit hair waste [28]. The above description shows that to obtain high solubility when ionic liquids are used, the dissolution temperature should be high.

Rabbit hair is a kind of protein fiber composed of 18 kinds of amino acids, and a total amino acids content of 96% [29]. Rabbit hair resources are rich, and more than 8000 tons of rabbit hair is produced annually in China [30,31]. However, the squama of rabbit hair is elongated and closely arranged, and has a small angle, thus has low friction coefficient, low crimpness, and uneven distribution of orthocortex and partial cortex, which can in turn cause low spinnability when mix with other fibers [32]. Additionally, the length of hair can vary among different rabbit species; therefore, a large amount of rabbit hair waste is produced during the spinning process every year, resulting in a severe waste of resources and major accumulation of environmental pollution. Therefore, the conversion of rabbit hair wastes into useful keratin materials has attracted increasing research attention. Rabbit hair has a large medullary layer; its structure is uniformly and hierarchically arranged, which is conducive to ultrasonic cavitation [33,34]. The aim of this study is to investigate the keratin extraction processes from rabbit hair, by combining measures of ultrasonic pretreatment and chemical methods. The structure and properties of the extracted keratin were characterized, demonstrating its biological compatibility, antioxidant activity and its self-assemblies characteristics.

## 2. Materials and Methods

### 2.1. Materials

Rabbit hairs were collected from German Angora rabbit warren (Gansu, China). Urea and Sodium bisulfite were purchased from Damao chemical reagent Co. Ltd. (Tianjin, China). Sodium dodecyl sulfate (SDS) was purchased from Usolf Co. Ltd. (Shenzhen, China). Acrylamide, N, N′-methylene-bisacrylamide, ammonium persulfate, pyrogallol, glycerin, methanol acetic acid, bromophenol blue and glycine were purchased from FUCHEN chemical reagent Co. Ltd. (Tianjin, China). Coomassie brilliant blue R-250 was purchased from Sigma-Aldrich (Shanghai, China). Tetramethylethylenediamine (TEMED), β-Mercaptoethanol and Tris-HCl Buffer were purchased from MACKLIN (Shanghai, China). BSA (0332-100G) was purchased from VWR (Westchester, PA, USA). Paraformaldehyde (BL539A) was purchased from BioSharp (Hefei, China). TritonX-100 (90002-93-1) was purchased from Biotopped (Beijing, China). Cell Counting Kit-8 (CCK-8 C0038) and DAPI (C1002) were obtained from Beyotime Institute of Biotechnology (Shanghai, China). Primary antibody (β-actin AF7018) and fluorescent secondary antibody (FITC S0006 Goat Anti-Rabbit IgG (H+L) Fluor594-conjugated) were purchased from Affinity Biosciences (Golden, CO, USA).

### 2.2. Preparation of Keratin from Rabbit Hair

To remove grease, rabbit hair was treated with petroleum ether at 75 °C for 3 h, and then treated with 50% ethanol at 70 °C for 2 h. After that, the hair was washed with distilled water and then subjected to ultrasonic treatment at 60 °Cat an oscillation frequency of 50 kHz. The clean, wet rabbit hair was then dried at 60 °C in an oven.

Two grams of ultrasonic-treated rabbit hair was immersed in 20 mL of mixed urea-sodium bisulfite-sulfate solvent, and the solution was heated and mechanically stirred until the rabbit hair was completely dissolved. The solution was filtered to remove the undissolved rabbit hair, and the obtained rabbit keratin solution was subsequently dialyzed in distilled water using a dialysis tube (with molecular cut off = 8–14 kDa) for 48 h to remove small molecules and salt formed in the reaction; during which, the distilled water was changed every 4 h. The dialyzed solution pH was the adjusted 4.0 (the isoelectric point of keratin) using hydrochloric acid. The rabbit keratin was obtained after centrifugation at 5000 r/min for 8 min. The obtained rabbit keratin was then freeze-dried and then ground to powder. Protein was stored at 4 °C before further use.

The extraction via sulfuretted step was carried out as follows: hydrophobic bonds and disulfide bonds were cleaved with urea and sodium bisulfite, respectively. Because the extraction efficiency can depend largely on the extraction conditions, the effects of various extraction conditions, including ratio of urea and sodium bisulfite, extraction time, temperature, and pH (Table 1) on the dissolution rate and the recovery of keratin were determined. Large micelles formed by SDS can effectively prevent the oxidation of sulfhydryl groups into disulfide bonds. The amount of SDS was maintained constant at 0.32 g because it has a low impact on the extraction efficiency of keratin [22].

### 2.3. Keratin Characterization

SDS-PAGE: The molecular weight distribution of keratin was determined by sodium dodecyl sulphate-polyacrylamide gel electrophoresis (SDS-PAGE) using a Mini-Protein Tetra system (Beijing LiuYi Biotechnology Co., LTD.), 10% separating gel, and 5% stacking gel, according to Laemmli’s method [35]. The electrophoresis was carried out at a voltage of 200 V, and the gel was stained with Coomassie brilliant blue R-250 and destained with a destaining solution containing 10% acetic acid and 10% methanol until a clear background was observed.

Amino acid analysis: Amino acid contents of rabbit hair and keratin were tested using the L8900 amino acid analyzer (Hitachi, Japan). The samples were hydrolyzed in 6 M hydrochloric acid (HCL) for 24 h at 110 °C under nitrogen atmosphere. Free amino acid residues were derivative with hydroxyl succinimidyl carbamate and eluted on a reversed-phase column. An Alliance High Performance Liquid Chromatograph (HPLC) (Shimadzu LC-20AT, Kyoto, Japan) was used; the eluate was detected at 254 nm. The quantitative amino acid composition was determined by external standard calibration (Amino Acid Standard H, Pierce). The fluorescence intensity was determined by alkaline hydrolyzed protein at the excitation wavelength of 280 nm and emission wavelength of 360 nm. The content of tryptophan in the sample was calculated according to the fluorescence intensity curve of standard tryptophan.

Solubility and recovery: The solubility (S) and the recovery (R) were calculated using Equations (1) and (2), respectively.

(1) $$S=\frac{w-w_{1}}{w}\times 100\%$$

(2) $$R=\frac{w_{2}}{w-w_{1}}\times 100\%$$

where *W* is the initial weight of rabbit hair, *W*_1_ is the weight of undissolved rabbit hair, and *W*_2_ is the weight of keratin.

Chemical structures of rabbit hair and keratin were characterized using a Fourier transform infrared (FT-IR) spectroscope operated in the transmission mode. The FTIR spectra were recorded at a wave number range of 4000 cm^−1^ to 400 cm^−1^, at a resolution of 4.0, and at 40 scans per sample. Raman spectra were obtained on a Renishaw in Via Microscope Raman. The laser excitation was provided with an argon ion laser operating at 10.2 mW of 633 nm output. Spectra were recorded between 2000 and 300 cm^−1^. CD spectra of farultraviolet light (180–250 nm) were acquired with Chirascan circular dichroism. Crystal structures of keratin extracted were determined by X-ray diffractometer (XRD, Brucker D8, Karlsruhe, Germany), operated using a Cu Kα radiation source. The samples were scanned at a 2α Bragg angle range of 5° to 60° at 0.02° step size and a scan speed of 3°/min. Thermal behaviors of rabbit hair and keratin were investigated using a thermal analyzer (TG-DSC, Netzsch STA 449F3, Selb, Germany). The phase change temperature and decomposition temperature were measured using a differential scanning calorimeter at a heating rate of 10 °C/min over a temperature range of 50 °C to 500 °C.

Dynamic Light Scattering (DLS): The particle size of the extracted keratin extracts at 2 mg/mL in water was measured using Zetasizer (NanoBrook, Malvern, UK) at 25 °C in triplicates.

Superoxide Anion Free Radical Scavenging In Vitro: We used pyrogallol auto-oxidation methods to assay oxygen scavenging properties as described by Lapenna et al. [36]. In the test, 4 mL of TRIS-HCL buffer (0.05 mol/L, pH = 8.2) was mixed with 1 mL of the sample (0.01%, 0.1%, 0.4%, 0.8%) and 1 mL of pyrogallol solution (0.2 mmol/L), and the absorbance (*A_i_*) was measured at 320 nm. the three reagents were preheated in advance in a water bath at 25 °C for 20 min. A mixture of 1 mL of deionized water with 4 mL of TRIS-HCL buffer and 1 mL of pyrogallol solution served as a blank. Its absorbance is denoted as *A*_0_. Considering that the sample absorbs UV light at wavelengths of 320 nm, the absorbance of the mixture solution without pyrogallol solution was studied (*A*_1_). The radical scavenging rate was calculated using Equation (3). Oxidation resistance became stronger as the scavenging rate increased.

(3) $$\mathrm{The}\;\mathrm{scavenging}\;\mathrm{rate}\;\left(\%\right)=\frac{A_{0}-A_{i}+A_{1}}{A_{0}}\times 100\%$$

Biocompatibility assay: The cytotoxicity of rabbit hair keratin was evaluated by using the LDH assay on freshly passaged L929 mouse fibroblasts cell lines in vitro cultures. The cells in good growth condition with a confluence of about 80% were prepared into single-cell suspension by removing the medium, washing with PBS, and digesting with trypsin. The cells were inoculated into 96-well plates with 500 cells/100 µL per well and incubated at 37 °C and 5% CO_2_ overnight. After 24 h of cell culture, different concentrations of keratin solution were added to the cells at 0, 1, 5, 10, 50, 100, 200, 500, and 1000 ng/ul, and treated for 1 and 2 days, respectively. 10 µL of CCK-8 was added to each well. The cells were further incubated at 37 °C for 2 h and the absorbance of the wells was measured at 450 nm. Wells without keratin solution served as the control group.

Immunofluorescence examination: The cells were soaked with PBS for 3 times, every 3 min, then, it was fixed with 4% paraformaldehyde for 15 min. The wells were washed with PBS 3 times and permeabilized with 0.1% Tritonx-100 for 20 min at room temperature. The soaked cells were sealed with 5% BSA (PBS preparation) for 1 h and then removed. Cells were incubated with primary antibody (β-actin) diluted with 5% BSA for overnight at 4 °C, washed with PBST and further incubated with diluted FITC for 2 h at 37 °C. Anti-fluorescence quenching agent containing DAPI was added and incubated in dark for 5 min. Images were observed and collected under a fluorescence microscope (Leica S/N479602).

## 3. Results and Discussion

### 3.1. Extraction of Keratin from Rabbit Hair

**Figure 1:**
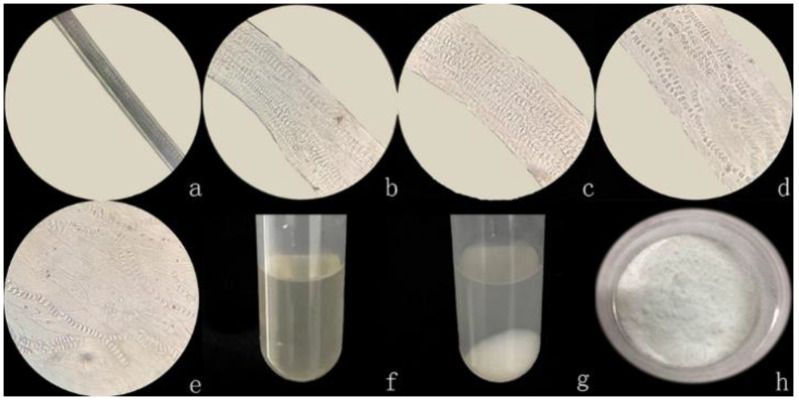
(**a**–**e**) Observation of the extraction process via light microscopy under different swelling times: (**a**) 0 h, (**b**) 0.5 h, (**c**) 1 h, (**d**) 2 h, (**e**) 3 h, and (**f**) Keratin solution (4.5 h). (**g**) Keratin sediment. (**h**) Keratin powder.

The degradation process of rabbit hair in the solvent system was observed under an optical microscope at 40x magnifications, as shown in Figure 1, solvent system first gets into the medullary layer of rabbit hair and cause the fiber to swell, and the fiber diameters increased with the increase of dissolution time. As shown, after 30 min of dissolution, the distance between the medulla gradually increased as the medulla laterally expanded along the fibers, and the color of the medullary layer was also gradually faded. The cavity shape was obviously observed when the dissolution time was 1 h. The systematic arrangement of the medulla became more legible, and the shape of the fiber changed from circle to sheet. At the dissolution time of 2 h, continuous segments of the medullary cavity were established, due to the rupture of the parietal as the swelling continued; nonetheless, the morphology of rabbit hair remained intact. After 3 h, the medulla was completely disintegrated, causing the entire fiber to burst, whereas after 4.5 h, most fibers were dissolved to form a keratin solution (Figure 1f). The medullary layer of rabbit hair is composed of multiple columns of medulla that are uniformly arranged along the fiber axis; and the number of this medullary layer increases as the fiber fineness increases. The medulla consists of regularly arranged rectangular cavities, which allow the solution to quickly penetrate into the cavity of the medulla. These cavities are interconnected through transverse septum materials, and each row of the medulla is connected by longitudinal septum materials. Unlike the cortical layer, the spacer material has an incompact microfiber-matrix structure [32]; thus, when the cavity is filled with a solution, the solution is more likely to react with the spacer material, causing the fiber to swell laterally and become flat rather than round. These data showed that the swelling of the medulla is an essential foundation of fiber dissolution and for extraction of keratin from protein fibers with rich medullary layers.

**Figure 2:**
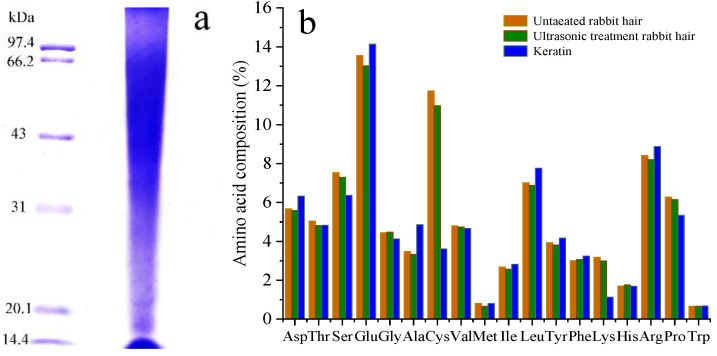
(**a**) SDS-PAGE of extracted rabbit hair keratin, (**b**) Amino acid composition (mole%).

The molecular weight of the extracted keratin was analyzed by SDS-PAGE (Figure 2a). The results revealed that the molecular weight of the extracted rabbit hair keratin is exhibited more dispersed band structure and some fragments had molecular weight concentrated in 15 kDa and 22 kDa, but the majority had a molecular weight ranging from 31 kDa to 94 kDa.

Figure 2b shows the amino acid composition of keratin, with respect to the amino acid composition of the fibers before and after ultrasound treatment. The content of various amino acids decreased in ultrasonic pretreatment fiber compared with untreated fiber. From this knowable, amino acids could be dissolved with ultrasonic treatment. The key of keratin extraction is to break the disulfide bond, then reduces the content of cystine and promotes fiber degradation. As shown in Figure 2b, the most evident change with respect to the original rabbit hair is the loss of cystine, the amount of cystine decreased from about 11.74% of the original rabbit hair to about 3.61% of the keratin. Besides, serine, lysine and line were reduced to some degree. Only the methionine and tryptophan content were quite similar in the samples.

### 3.2. Effect of Extraction Conditions on Extraction Efficiency

**Figure 3:**
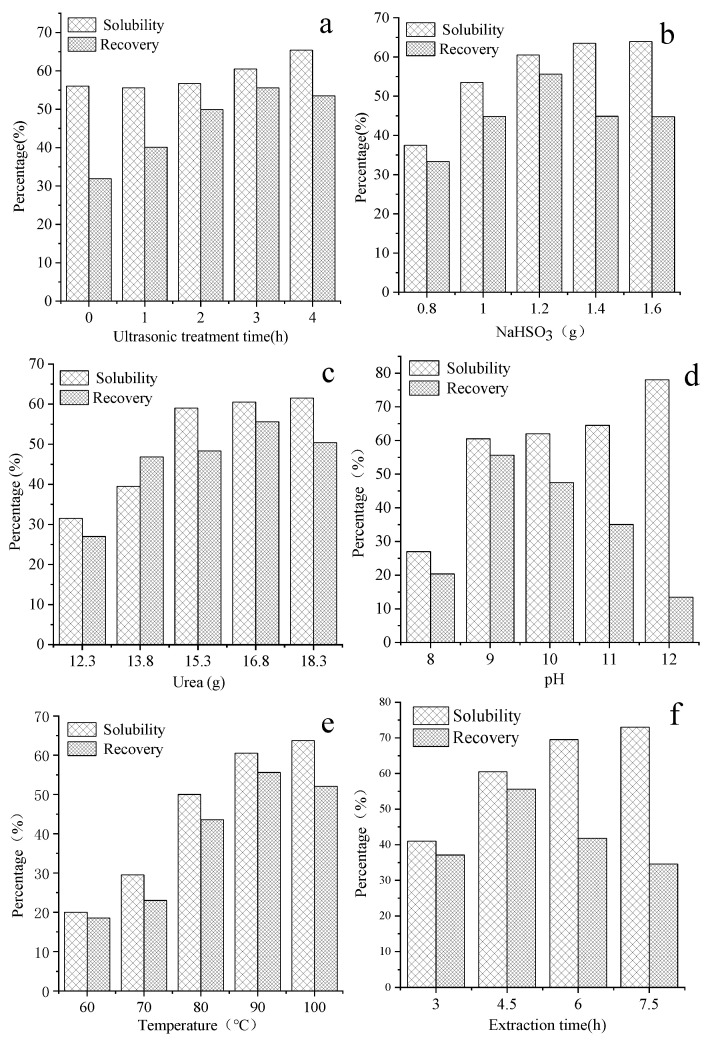
Effect of various factors on the rabbit hair keratin extraction using the ultrasonic-assisted reducing agent-based method: (**a**) Effect of ultrasonic treatment times, (**b**) Effect of sodium bisulfite dose, (**c**) Effect of urea dose, (**d**) Effect of Ph, (**e**) Effect of extraction temperature, and (**f**) Effect of extraction time.

Figure 3a shows the effect of ultrasonic treatment time on solubility and recovery. The solubility of rabbit hair was slightly changed when the ultrasonic treatment time was less than 2 h; but the solubility significant increased with increasing ultrasonic treatment time after 2 h. The overall recovery of ultrasonic-treated rabbit hair was higher than that of untreated rabbit hair. The recovery of keratin reached the maximum value when the ultrasonic treatment time was 3 h, the recovery was about 74% higher that than of the untreated rabbit hair. Additionally, the ultrasonic treatment can not only promote the dissolution of fiber, but also ensure the high recovery of keratin powder. Rabbit hair has a cylindrical shape. The degree of dissolution of the interior and exterior structure of the fibers is different because the dissolution process takes place from the exterior to the interior. An ultrasonic wave is employed to pretreat the fiber so that its structure becomes loosen, and the solvent can in turn quickly penetrate into the fiber to increase its solubility. This can then reduce the difference between the solubility of the interior and the exterior of the hair, causing keratin to have a narrow molecular weight distribution, which is conducive to the formation of keratin precipitates. The ultrasonic treatment is simple method that can be used to extract keratin and to increase the solubility of rabbit hair. At the initial stage of dissolution, the treatment can decrease the crystallinity of rabbit hair, facilitating the solvent to rapidly penetrate into the interior structure of the fiber and break its disulfide bond, as has been previously reported [37].

As shown in reaction (1), with sodium bisulfite as a reducing agent, disulfide bonds (s-s) in rabbit hair were cleaved causing the hair to lose its 3D structure, which then released cysteine and cysteine-S-sulfonate [38]. The solubility curve of rabbit hair and the recovery curve of keratin obtained at different sodium bisulfite doses are illustrated in Figure 3b.

(R1) $$\begin{array}{l} P-S-S-P+NaHSO_{3}\to P-SSO_{3}Na+P\prime -SH\\ Rabbithair\;\mathrm{Sodiumbisulfite}\;\mathrm{Cysteine-S-sulphonate}\;\mathrm{Cysteineresidues} \end{array}$$

According to the results, the solubility of rabbit hair increased with increasing dose of sodium bisulfite; however, at a sodium bisulfite dose of more than 1.2 g, the increase became less accentuated. This is due to the fact that with the increase of sodium bisulfite dose, most disulfide bonds are broken, in turn causing further dissolution of rabbit hair. With increasing sodium bisulfite dose, the recovery of keratin first increased and reached the maximum value of 55.6% when the sodium bisulfite was 1.2 g; thereafter, the recovery decreased. This is due to that there is limited destruction of disulfide bonds at a low dose of sodium bisulfite. This solution, which also contains many long macromolecular chains, was unable to precipitate at isoelectric point; this resulted in low recovery of keratin. With the increase of sodium bisulfite, long macromolecular chains were broken into shorter macromolecular chains and single molecules. The keratin recovery decreased when the dose of sodium bisulfite was increased to above 1.2 g. This is due to the fact that most disulfide bonds were broken, and the degradation of keratin resulted in polypeptides with low molecular weights that could permeate through dialysis tube [39].

Urea can modify the structure of rabbit hair fiber by breaking hydrophobic bonds between the molecules, causing the swelling of the fiber and its adsorption performance to increase. Nonetheless, the solubility of rabbit hair and recovery of keratin obtained under different doses of urea is shown in Figure 3c. As illustrated, with increasing urea dose, the solubility rapidly increased reaching its maximum value at 15.3 g of urea, but thereafter more slowly increased until reaching a steady state. The recovery of keratin first increased, but decreased thereafter; the highest recovery of keratin was 55.6% at the urea doses of 16.8 g. The increased dose of urea can lead to increased degree of swelling of rabbit hair, which can facilitate the reducing agent and the rabbit hair to fully interact with each other. At the same time, urea can be broken down in water into ammonia that causes the increase of pH, which in turn leads to the hydrolysis of peptide bonds to produce more low-molecular weight peptides and amino acids.

As illustrated in Figure 3d, with increasing pH, the solubility rapidly increased until reaching a nearly constant value. In contrast, when the pH was increased to 12, the solubility further increased rapidly. The recovery of keratin was highest at pH 9. In the isoelectric point precipitation of keratin, the pH values of keratin solution were respectively adjusted to 2.5, 4, and 5.5. Precipitation was almost unobservable in the keratin solution extracted at pH 12, when the isoelectric point was either 2.5 or 5.5. By contrast, a very low amount of precipitate was observed in the solution extracted at pH 9, considering that the isoelectric point is 5.5. Thus, to improve the solubility, the macromolecular chain of the fiber should be further broken into small peptides and other small molecules; this can affect the recovery of keratin extracted at its isoelectric-point.

The effect of extraction temperature is illustrated in Figure 3e. Based on the results, as the temperature increased, the solubility of rabbit hair significantly increased, whereas the recovery of keratin first increased and then decreased. The recovery of keratin was highest when the temperature was 90 °C. This result indicates that the dissolution of rabbit hair is favorable at high temperatures, and with increased dissolution rate, smaller polypeptide molecules are produced, which is not conducive to the recovery of keratin at its isoelectric point.

The effect of extraction time on keratin extraction was assessed, and the result is depicted in Figure 3f. The fiber in Figure 1 started to dissolve after 3 h; therefore, the extraction times selected for the experiments were between 3 and 7.5 h. It was found that a long extraction time was conducive to the dissolution of the fiber, but the recovery rate of keratin decreased significantly when the extraction time exceeded 4.5 h, which indicates that keratin would be decomposed after a long period of time.

The efficiency of extraction is a critical factor affecting the production of keratin from fiber at the industrial scale. Based on the above results, the extraction should be carried out under the following conditions: ultrasonic treatment time of 3 h, 1.2 g of sodium bisulfite, 16.8 g of urea, 0.32 g of SDS, 90 °C, 4.5 h, and pH 9.

### 3.3. Molecular Structure Characterization of Keratin

**Figure 4:**
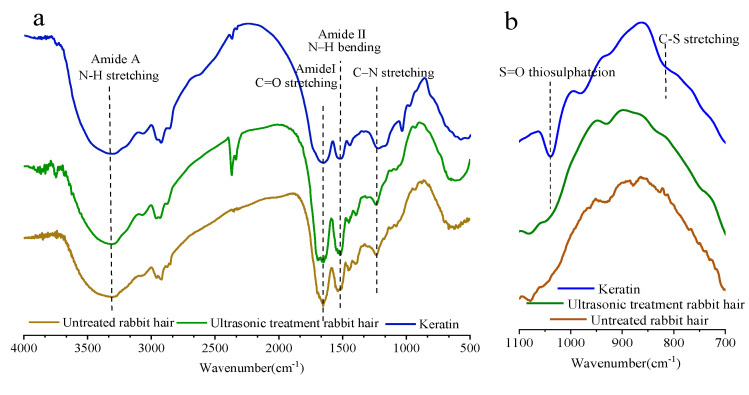

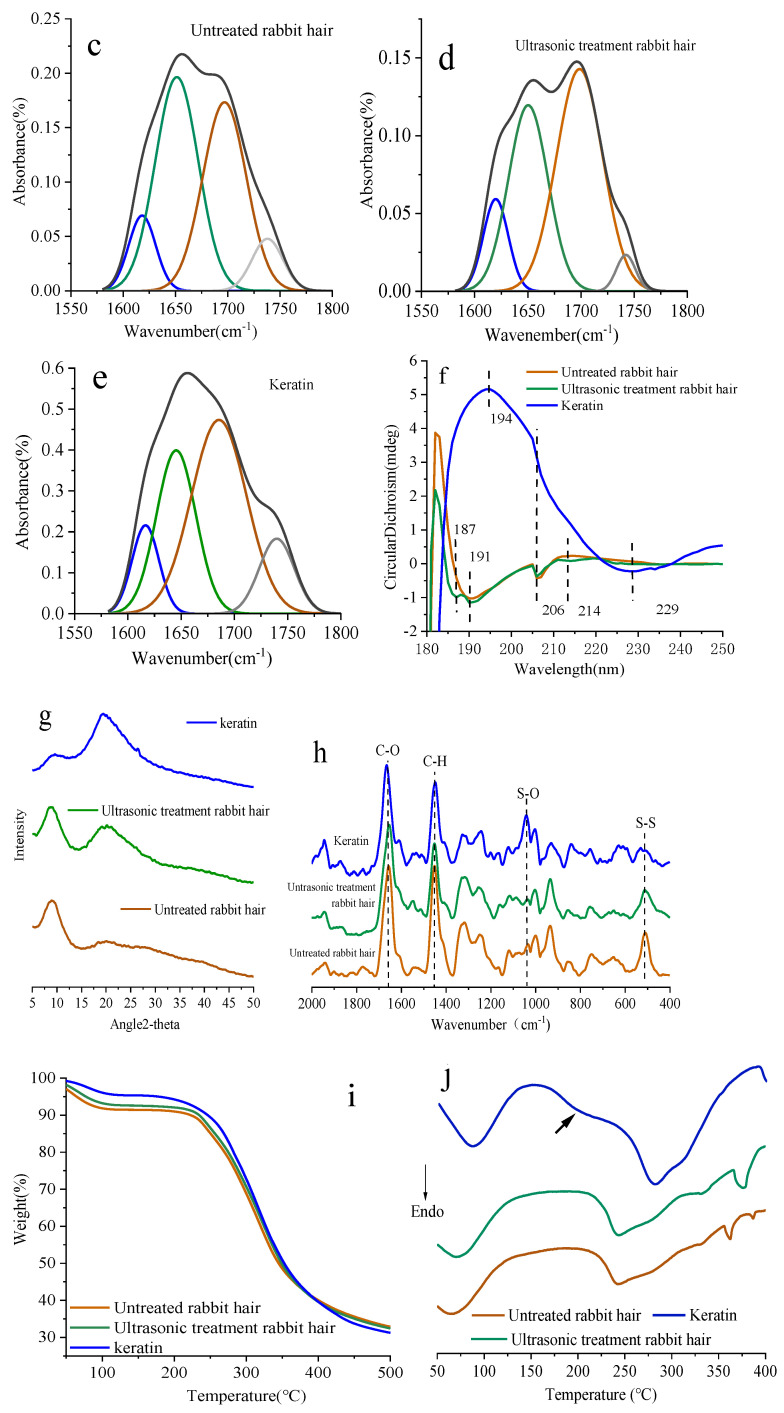
Structural characterization of extracted keratins: (**a**–**e**) FTIR spectra, (**f**) Circular dichroism (CD) spectrum, (**g**) X-ray diffraction (XRD), (**h**) Roman spectra, (**i**) TGA, (**j**) DSC.

The FTIR spectra of untreated and ultrasonic-treated rabbit hair, and keratin extracted at pH 9 are shown in Figure 4. Figure 4a depicts that the FTIR spectra is the typical FTIR spectra of keratin, as can be observed by the obvious characteristic absorption peaks of amide A, amide I, amide II, and amide III. The broad band of amide A observed at approximately 3300 cm^−1^ can be attributed to the stretching vibrations of -O-H and -N-H. The amide I band observed at 1600–1700 cm^−1^ is due to the stretching vibration C=O, while the amide II band appeared at approximately 1500 cm^−1^ is related to the out-plane bending vibration of N–H. The amide III band appeared between 1150 and 1200 cm^−1^ is caused by the in-phase stretching vibration of C–N. Similar spectra have also been reported in relevant literature [40,41,42]. The enlarged FTIR spectra at a frequency range of 700 and 1100 cm^−1^, which are sensitive to disulfide bonds, is shown in Figure 4b. The characteristic absorption peaks between 700 cm^−1^ and 1100 cm^−1^ of the three samples were significantly different. The keratin sample exhibited obvious characteristic absorption peak at about 1030 cm^−1^ and 810 cm^−1^ compared to rabbit hair, especially for that at about 1030 cm^−1^, which is due to the symmetric stretching vibration of S=O in sulfonated cysteine residue [43]. The intensity of the absorption peak at 1030 cm^−1^ of keratin noticeably increased, suggesting that increasing number of disulfide bond was reduced to form cysteic acid. The characteristic absorption peak at approximately 800 cm^−1^ is caused by the stretching mode of C-S sulfonate [44]. The adsorption band of amide I located between 1600 and 1700 cm^−1^ is correspondent to the secondary structures of the protein, which is particularly sensitive to the supramolecular structure; this band is traditionally used to calculate the contents of α-helix and β-sheet structures [45,46]. In this work, to study the changes of the secondary structures of rabbit hair and keratin, the amide I band was resolved by the Gaussian function, and the results are shown in Figure 4c–e and Table 2. According to the results, we observed that high peak area of α-helix (located at 1651 cm^−1^), moderate peak area of random coil, and relatively low peak area of β-sheet were observed in the untreated rabbit hair. The peak area of α-helix of the ultrasonic-treated rabbit hair decreased, while that of random coil and β-sheet increased, compared with those of the untreated rabbit hair. This shows that the ultrasonic treatment can reduce the crystallinity of the rabbit hair to some extent; however, the treatment appears to insufficiently destroy the chemical structure of the fiber. On the other hand, different trends were observed in keratin: the peak of α-helix structure disappeared in keratin samples, and as a result, the contents of β-sheet and random coil significantly increased. Furthermore, the extracted keratin contained a higher content of terminal COOH groups compared to rabbit hair. It is likely that the dissolution process caused the destruction of the protein secondary structures by disrupting the disulfide and hydrogen bonds.

The secondary structure of rabbit hair keratin extracts was further determined from the CD spectra (Figure 4f). The obvious negative Cotton effect at 190 nm and 206 nm of the untreated rabbit hair can be attributed to the typical α-helix structure. The CD spectra of the ultrasonic-treated rabbit hair has new negative Cotton peak at 187 nm and 214 nm, which corresponds to β-turn and β-sheet structure, respectively. The circular dichromatography characteristics of the extracted keratin solution are different from rabbit hair. The negative Cotten peak at 190 nm disappeared completely, and the negative Cotten peak at 206 weakened and was replaced by the positive Cotten peak at 194 nm and the negative Cotten peak at 229 nm. This is a typical characteristic of the conformational transformation of keratin molecules from α-Helix to β-sheet and β-turn.

The wide-angle X-ray diffraction spectrogram of the rabbit hair fiber and keratin are presented in Figure 4g. The untreated rabbit hair exhibited a strong diffraction peak of the α-helix structure at 9°and a broad and weak diffraction peak of the β-sheet structure at 20°. Compared with that of the untreated rabbit hair, the intensity of the peak at 9° of the ultrasonic-treated rabbit hair decreased, while that of the peak at 20° increased, indicating that the ultrasonic treatment could destroy the structure due to the stress and strain concentrations in some areas of the rabbit hair, which is consistent with the FTIR results [37].The keratin sample exhibited a strong diffraction peak of the β-sheet structure at 20° and a weak diffraction peak of the α-helix structure at 9°, indicating that the chemical dissolution can convert the α-helix structure of rabbit hair into the β-sheet structure. Similar changes have been observed in keratin extracted from rabbit hair using a mixture of chloride/oxalic acid (a deep eutectic solvent) [28]. The crystallinity of the three kinds of samples, including untreated rabbit hair, ultrasonic-treated rabbit hair, and keratin, was estimated and found to be 43.53%, 33.51%, and 23.99%, respectively.

The Raman spectra between the rabbit hair and the samples treated with ultrasonic and keratin are observed in the 400–2000 cm^−1^ range (Figure 4h), where could be assigned to S-S bond (512 cm^−1^), the C-O bonds (1655 cm^−1^), and the C-H bonds (1450 cm^−1^) [47,48]. The absorbance peaks of investigated samples showed almost the same absorption position. Disulfide bond cleavage is a key part of keratin dissolution. The intensities of S-S bond in the ultrasonic-treated rabbit hair slightly reduced. In the spectra obtained from the keratin no clear evidence of this disulfide vibration is observed suggesting that a large of the disulfide bonds in the keratin have been cleaved, while the S-O band assigned to cysteic acid was observed at 1040 cm^−1^ [49].

The thermal stability of the rabbit hair and the extracted keratin were investigated by TG-DSC, and the two-stage decomposition process was observed in all cases (Figure 4i). A small weight loss was observed in the first stage, and this loss could be due to the release of absorbed water. It is worth noting that water evaporation in the keratin took place at a higher temperature (85 °C) than that in the rabbit hair (70 °C). This may be due to the low degree of crystallization and high surface area of keratin, as a result, it is easier for water to combine with hydrophilic groups to form bound water. The second weight loss, which was large, could be observed at a temperature range of 200 °C to 375 °C, due to the melting and decomposition of the materials [18,28].

The DSC thermogram for the ultrasonic-treated rabbit hair (Figure 4j) shows an endothermic peak around 242 °C, with a trace of α-helix disordering and fiber melting, similarly to the DSC thermogram of the untreated rabbit hair. The endothermic peak in the extracted keratin was broadened and shifted to a lower temperature range (200–300 °C) compared with that is the rabbit hair, due to that the breakage of disulfide bonds may occur during the keratin extraction process, which in turn cause the weakening of the secondary structures of proteins, resulting in the formation of disordered structures [26,27]. The DSC curve of keratin showed the bimodal endothermic peak at 202 °C and 285 °C. This observation is in agreement with the highest weight loss observed in TGA. Some studies [2] have described that the endothermic peak at 202 °C is caused by small-molecular-weight keratin fractions, which have low thermal stability. The endothermic peak at 285 °C is corresponded to the melting/degradation of the intermacrofibrillar matrix of keratin containing high sulfur content.

### 3.4. Property Characterization of Keratin

**Figure 5:**
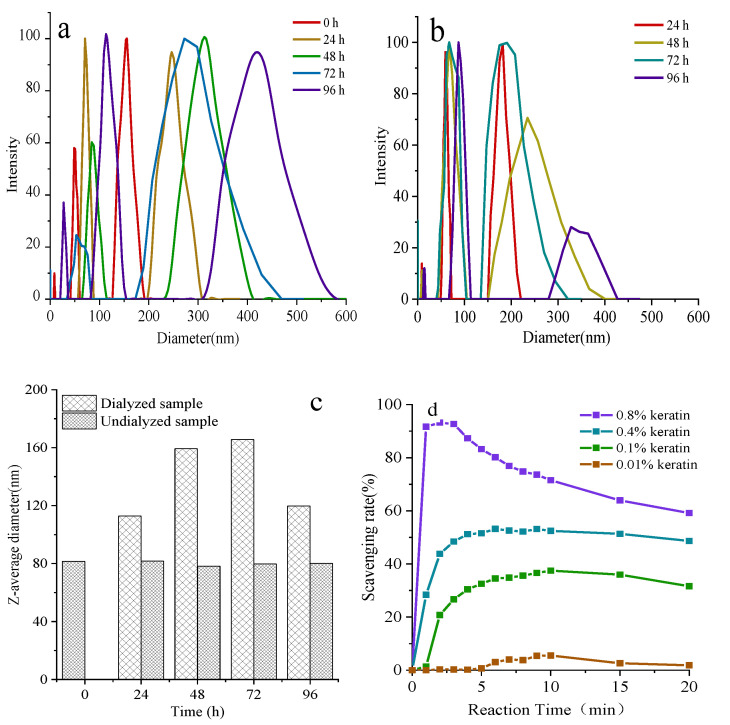
Dynamic light scattering of keratin in ultrapure water with generated plots measuring intensity%, (**a**) Dialysis samples at different times (**b**) Undialyzed sample (**c**) Average diameter of the two samples (**d**) Superoxide anion free radical scavenging test.

Keratin solution profiles reconstituted in ultrapure water are shown by Figure 5. The keratin powder reconstructed in ultrapure water showed the greatest dynamic instability in solution, the particle diameter of keratin increased with the increase of dialysis time (Figure 5a). The initial particle diameter of keratin solution is about 80 nm, and after 96 h of dialysis with deionized water, the particle diameter increased to about 165 nm (Figure 5c). In contrast, the particle diameter of keratin without dialysis was placed 4 °C for 96 h has little changes (Figure 5b). Therefore, large keratin particles can be formed by assembly with a balance between intermolecular hydrophobic attractions as the concentration of urea in keratin solution decreased during dialysis [50].

Studies have demonstrated that the antioxidant properties of keratin are due to its high cysteine content [51]. Therefore, the ability of keratin to scavenge superoxide anion free radicals was examined in vitro. As shown in Figure 5d, keratin quickly eliminated free radicals within 5 min, and the efficiency of free radical scavenging increased obviously with the increase of keratin concentration. Free radical scavenging rate was 5% when keratin concentration was 0.01%, however, it can be achieved above 70% when the concentration of keratin rises to 0.8%, which was almost equivalent to that for 1% wool keratin [4].

**Figure 6:**
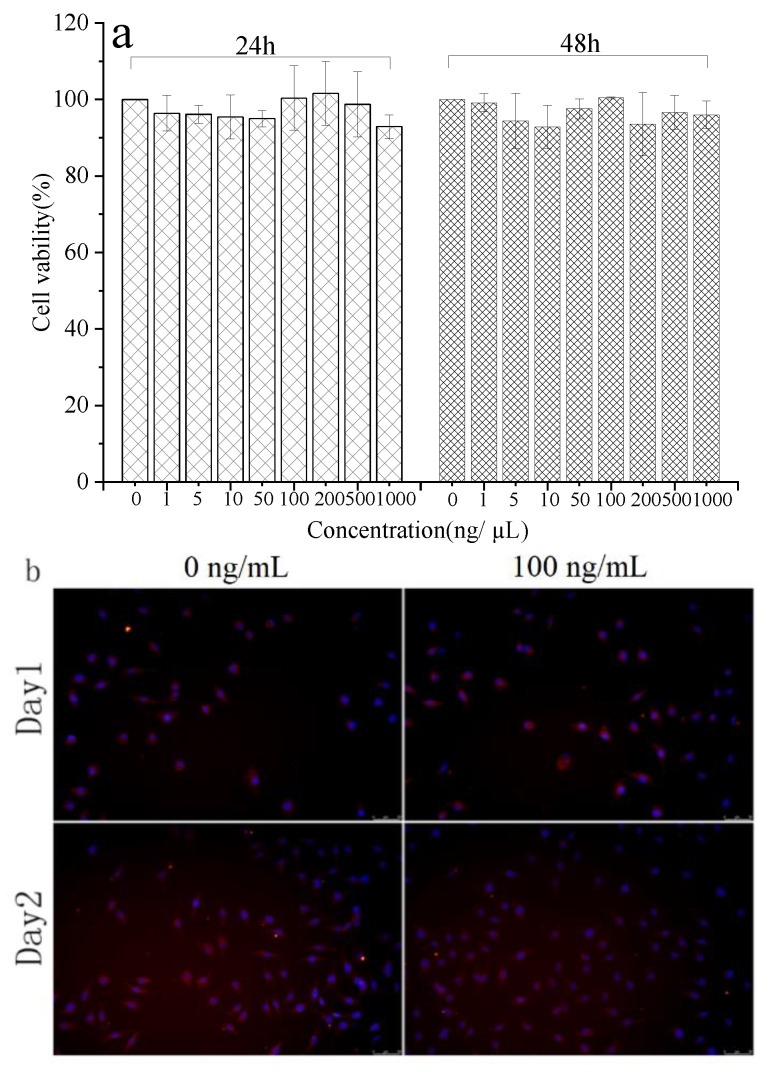
Bioactivities characterization of extracted keratins: (**a**) CCK-8 test, (**b**)Fluorescent staining with β-actin/DAPI of L929 mouse fibroblasts with keratin (100 ng/mL) after 1 days and after 2 days of cultivation. The cells were stained for living cells (red) and nuclei (blue). Each group was performed in triplicate and the value was expressed as mean ± variance.

The biocompatibility of medical materials is a crucial factor that must be considered prior. Therefore, it is critically important to test the cell–material interactions in vitro to prove the potential applicability of materials in human medicine. Cytotoxicity of rabbit hair keratin with different concentrations was evaluated in vitro. The results of the MTT assay (Figure 6a) demonstrated that soluble keratin from rabbit hair is not cytotoxic during 2 days of culture, and the number of cells treated with keratin solution changed very little with results similar to the control sample. Figure 6b shows the L929 mouse fibroblasts with keratin after 1 and 2 days of incubation. the cell shape and membrane integrity were characterized by β-actin staining, furthermore, the cells were also stained with DAPI to study the integrity of the nucleus [52]. The results of morphological evaluation furthermore demonstrated that the integrity of cell membrane and the nucleus were maintained after adding keratin.

## 4. Conclusions

Waste resulted from rabbit hair with poor spinnability has caused environmental problems; thus, it is particularly important to fully utilize rabbit hair with a high keratin content. In this study, ultrasonic-assisted reducing agent-based extraction method was developed to extract keratin from rabbit hair. We showed that it is feasible to extract keratin from rabbit hair using the developed extraction technique. Under the optimum extraction conditions, the extraction rate of 55.6% was achieved, and the extracted keratin could retain most of the protein skeleton, despite the breakage of disulfide and hydrogen bonds. Moreover, the infinite swelling of the medullary layer in the rabbit hair caused by the reducing agent is the primary cause of fiber breakage and degradation during dissolution. The extracted keratins showed excellent biocompatibility, antioxidizability and Self-assembly.

## Figures and Tables

**Table 1:** Different extraction conditions.

Ultrasonic Treatment Time/h	Extraction Time/h	NaHSO_3_/g	Urea Dose/g	Temperature/°C	pH
0	3	0.8	12.3	100	8
1	4.5	1	13.8	90	9
2	6	1.2	15.3	80	10
3	7.5	1.4	16.8	70	11
4	-	1.6	18.3	60	12

**Table 2:** Contents of α-helix and β-sheet in various samples.

Sample	Wavenumber (cm^−1^)	Assignment	Content (%)
Untreated rabbit hair	1618	β-sheet	9.66
	1651	α-helix	43.73
	1697	random coil	38.98
	1738	Terminal COOH	7.63
Ultrasonic treatment rabbit hair	1620	β-sheet	11.14
	1650	α-helix	35.63
	1699	random coil	49.86
	1742	Terminal COOH	3.37
Keratin	1617	β-sheet	10.72
	1645	random coil	29.06
	1686	β-sheet/random coil	48.36
	1740	Terminal COOH	11.86

## Data Availability

Data is contained within the article.

## References

[1] Shavandi A., Silva T.H., Bekhit A.A., Bekhit E.D. (2017). Keratin: Dissolution, extraction and biomedical application. Biomater. Sci..

[2] Vasconcelos A., Freddi G., Cavaco-Paulo A. (2008). Biodegradable materials based on silk fibroin and keratin. Macromolecules.

[3] Wang S., Wang Z., Foo S.E.M., Tan N.S., Yuan Y., Lin W., Zhang Z., Ng K.W. (2015). Culturing fibroblasts in 3D human hair keratin hydrogels. ACS Appl. Mater. Interfaces.

[4] Su C., Gong J.S., Ye J.P., He J.M., Li R.Y., Jiang M., Geng Y., Zhang Y., Chen J.H., Xu Z.H. (2020). Enzymatic extraction of bioactive and self-assembling wool keratin for biomedical applications. Macromol. Biosci..

[5] Tachibana A., Kaneko S., Tanabe T., Yamauchi K. (2005). Rapid fabrication of keratin-hydroxyapatite hybrid sponges toward osteoblast cultivation and differentiation. Biomaterials.

[6] Rouse J.G., Van Dyke M.E. (2010). A review of keratin-based biomaterials for biomedical applications. Materials.

[7] DeFrates K.G., Robert M., Julia B., Guowei L., Thomas M., Vince B., Xu H. (2018). Protein-based fiber materials in medicine: A review. Nanomaterials.

[8] Kornillowicz-Kowalska T., Bohacz J. (2011). Biodegradation of keratin waste: Theory and practical aspects. Waste Manag..

[9] Bhavsar P., Zoccola M., Patrucco A., Montarsolo A., Mossotti R., Rovero G., Giansetti M., Tonin C. (2016). Superheated water hydrolysis of waste wool in a semi-industrial reactor to obtain nitrogen fertilizers. ACS Sustain. Chem. Eng..

[10] Salminen E., Rintala J. (2002). Anaerobic digestion of organic solid poultry slaughterhouse waste-a review. Bioresour. Technol..

[11] Nakamura A., Arimoto M., Takeuchi K., Fujii T. (2002). A rapid extraction procedure of human hair proteins and identification of phosphorylated species. Biol. Pharm. Bull..

[12] Carolina C.A., Ricardo F., Ana P., Manuela A., Carlos D.P., Raquel M.A., Pintado M.E. (2018). Novel eco-friendly method to extract keratin from hair. ACS Sustain. Chem. Eng..

[13] Zoccola M., Aluigi A., Patrucco A., Vineis C., Forlini F., Locatelli P., Sacchi M.C., Tonin C. (2012). Microwave-assisted chemical-free hydrolysis of wool keratin. Text. Res. J..

[14] Zhang Y., Zhao W., Yang R. (2015). Steam flash explosion assisted dissolution of keratin from feathers. ACS Sustain. Chem. Eng..

[15] Blackburn S., Lee G.R. (1956). The reaction of wool keratin with alkali. Biochim. Biophys. Acta.

[16] Tsuda Y., Nomura Y. (2014). Properties of alkaline-hydrolyzed waterfowl feather keratin. Anim. Sci. J..

[17] Aluigi A., Zoccola M., Vineis C., Tonin C., Ferrero F., Canetti M. (2007). Study on the structure and properties of wool keratin regenerated from formic acid. Int. J. Biol. Macromol..

[18] Zhang J., Li Y., Li J.S., Zhao Z., Liu X., Li Z., Han Y.X., Hu J.Y., Chen A.Z. (2013). Isolation and characterization of biofunctional keratin particles extracted from wool wastes. Powder Technol..

[19] Fan J., Yu W.D. (2012). High yield preparation of keratin powder from wool fiber. Fiber Polym..

[20] Timmons S.F., Blanchard C.R., Smith R.A. (2000). Method of Making and Cross-Linking Keratin-Based Films and Sheets.

[21] Goddard D.R., Michaelis L. (1935). Derivatives of keratin. J. Biol. Chem..

[22] Poole A.J., Lyons R.E., Church J.S. (2011). Dissolving feather keratin using sodium sulfide for bio-polymer applications. J. Polym. Environ..

[23] Katoh K., Tanabe T., Yamauchi K. (2004). Novel approach to fabricate keratin sponge scaffolds with controlled pore size and porosity. Biomaterials.

[24] Xu H., Ma Z., Yang Y. (2014). Dissolution and regeneration of wool via controlled disintegration and disentanglement of highly crosslinked keratin. J. Mater. Sci..

[25] Li S.T., Zhang Y., Zhang H., Zhang R. (2017). Extraction of keratin from rabbit hair using L- cystein as reductive agent. Fine Chem..

[26] Idris A., Vijayaraghavan R., Rana U.A., Fredericks D., Patti A.F., Macfarlane D.R. (2013). Dissolution of feather keratin in ionic liquids. Green Chem..

[27] Ghosh A., Clerens S., Deb-Choudhury S., Dyer J.M. (2014). Thermal effects of ionic liquid dissolution on the structures and properties of regenerated wool keratin. Polym. Degrad. Stab..

[28] Wang D., Yang X.H., Tang R.C., Yao F. (2018). Extraction of keratin from rabbit hair by a deep eutectic solvent and its characterization. Polymers.

[29] Li S.T. (2017). Extraction of L-Arginine from Rabbit Hair. Master’s Thesis.

[30] Qian B.Q. (2017). The present situation and development prospect of rabbit hair. Chin. J. Rabbit Farm..

[31] Guzin K.Y. (2014). Angora rabbit fiber production in the world and Turkey. Am. J. Mater. Eng. Technol..

[32] Yan H.J., Yu X.F. (1988). Study on the structure and properties of rabbit hair. J. Text. Res..

[33] Yan S.T. (1991). A review of the structure and properties of rabbit hair. China Fiber Insp..

[34] Antunes E., Cruz Célia F., Azoia N.G., Cavaco-Paulo A. (2015). The effects of solvent composition on the affinity of a peptide towards hair keratin: Experimental and molecular dynamics data. RSC Adv..

[35] Laemmli U.K. (1970). Cleavage of structural proteins during the assembly of the head of bacteriophage T4. Nature.

[36] Lapenna D., Gioia S.D., Mezzetti A., Ciofani G., Cuccurullo F. (1995). Aminophylline: Could it act as an antioxidant in vivo?. Eur. J. Clin. Investig..

[37] Yuan J.G., Qian W.W., Zhu H.J., Wang Q., Fan X.R., Wang P., Li Z.R. (2013). Influence of ultrasonic treatment on structure of wool fiber. Tex. Dye. Finish. J..

[38] Yao J.B. (2003). Application and Preparation of Wool’s Keratin Solution. Ph.D. Thesis.

[39] Na Ayutthaya S.I., Tanpichai S., Wootthikanokkhan J. (2015). Keratin extracted from chicken feather waste: Wxtraction, preparation, and structural characterization of the keratin and keratin/biopolymer films and electrospuns. J. Polym. Environ..

[40] Zhang Y., Li S.T., Zhang H., Zhang Z.L. (2017). Structural characterization and application of rabbit whiting. J. Text. Res..

[41] Moore K.E., Mangos D.N., Slattery A.D., Raston C.L., Boulos R.A. (2016). Wool deconstruction using a benign eutectic melt. RSC Adv..

[42] Sharma S., Gupta A., Saufi B.T.C.S.M., Gek Kee C.Y., Poddar P.K. (2017). Dissolution and characterization of biofunctional keratin particles extracted from chicken feathers. IOP Conf. Ser. Mater. Sci. Eng..

[43] Erra P., Gómez N., Dolcet L.M., Juliá M.R., Lewis D.M., Willoughby J.H. (1997). FTIR analysis to study chemical changes in wool following a sulfitolysis treatment. Text. Res. J..

[44] Ramachandran E., Natarajan S. (2004). Crystal growth of some urinary stone constituents: III. In-vitro crystallization of L-cystine and its characterization. Cryst. Res. Technol..

[45] Aluigi A., Tonetti C., Rombaldoni F., Puglia D., Fortunati E., Armentano I., Santulli C., Torre L., Kenny J.M. (2014). Keratins extracted from Merino wool and Brown Alpaca fibres as potential fillers for PLLA-based biocomposites. J. Mater. Sci..

[46] Cardamone J.M. (2010). Investigating the microstructure of keratin extracted from wool: Peptide sequence (MALDI-TOF/TOF) and protein conformation (FTIR). J. Mol. Struct..

[47] Wojciechowska E., Wochowicz A., Weseucha-Birczyńska A. (1999). Application of Fourier-transform infrared and Raman spectroscopy to study degradation of the wool fiber keratin. J. Mol. Struct..

[48] Mi X., Li W., Xu H., Mu B., Yang Y. (2020). Transferring feather wastes to ductile keratin filaments towards a sustainable poultry industry. Waste Manag..

[49] Kuzuhara A., Hori T. (2013). Analysis of heterogeneous reaction between reducing agents and keratin fibers using Raman spectroscopy and microspectrophotometry. J. Mol. Struct..

[50] Lin D., Lin W., Gao G.Z., Zhou J.W., Chen T.B., Ke L.J., Rao P.F., Wang Q. (2020). Purification and characterization of the major protein isolated from Semen Armeniacae Amarum and the properties of its thermally induced nanoparticles. Int. J. Biol. Macromol..

[51] Fontoura R., Daroit D.J., Corrêa A.P.F., Moresco K.S., Brandelli A. (2018). Characterization of a novel antioxidant peptide from feather keratin hydrolysates. N. Biotechnol..

[52] Loannou Y.A., Chen F.W. (1996). Quantitation of DNA fragmentation in apoptosis. Nucl. Acids Res..

